# Genetic and Biological Effects of *SLC12A3*, a Sodium-Chloride Cotransporter, in Gitelman Syndrome and Diabetic Kidney Disease

**DOI:** 10.3389/fgene.2022.799224

**Published:** 2022-05-03

**Authors:** Nan Li, Harvest F. Gu

**Affiliations:** ^1^ Department of Endocrinology, Jiangsu Province Hospital of Traditional Chinese Medicine, The Affiliated Hospital of Nanjing University of Chinese Medicine, Nanjing, China; ^2^ Laboratory of Molecular Medicine, School of Basic Medicine and Clinical Pharmacy, China Pharmaceutical University, Nanjing, China

**Keywords:** diabetic kidney disease, genetic variant, gitelman syndrome, SLC12a3, sodium and chloride reabsorption

## Abstract

The *SLC12A3* (Solute carrier family 12 member 3) gene encodes a sodium-chloride cotransporter and mediates Na^+^ and Cl^−^ reabsorption in the distal convoluted tubule of kidneys. An experimental study has previously showed that with knockdown of zebrafish ortholog, slc12a3 led to structural abnormality of kidney pronephric distal duct at 1-cell stage, suggesting that *SLC12A3* may have genetic effects in renal disorders. Many clinical reports have demonstrated that the function-loss mutations in the *SLC12A3* gene, mainly including Thr60Met, Asp486Asn, Gly741Arg, Leu859Pro, Arg861Cys, Arg913Gln, Arg928Cys and Cys994Tyr, play the pathogenic effects in Gitelman syndrome. This kidney disease is inherited as an autosomal recessive trait. In addition, several population genetic association studies have indicated that the single nucleotide variant Arg913Gln in the *SLC12A3* gene is associated with diabetic kidney disease in type 2 diabetes subjects. In this review, we first summarized bioinformatics of the *SLC12A3* gene and its genetic variation. We then described the different genetic and biological effects of *SLC12A3* in Gitelman syndrome and diabetic kidney disease. We also discussed about further genetic and biological analyses of *SLC12A3* as pharmacokinetic targets of diuretics.

## Introduction

As the urine-producing organs, kidneys are vital in maintaining normal body functions because they are responsible for maintaining the balance of water, electrolytes, and the homeostasis of the internal environment through their filtration and reabsorption (Wallace, 1998). The function of kidneys is related with age. In general speaking, kidneys reach full functionality after the age of 5 years and suffer a slow and progressive decline in their regulatory range from the age of 20 years. Features of renal aging include several functional alterations, such as reduction of glomerular filtration rate, Na^+^ reabsorption, K^+^ secretion, vitamin D3 synthesis, titratable acid excretion, responsiveness to hormones, and regulatory flexibility (Gekle, 2017; Hommos et al., 2017). For the whole lifetime, a human being’s survival depends upon the crucial functions and processes performed by the kidneys (Jourde-Chiche et al., 2019). Renal disorders such as Gitelman syndrome (GS, presented in the patients after 6 years old), diabetic kidney disease (DKD, the patients are adults) may strike anyone at any age and at any time (Chevalier, 2019; Akhtar et al., 2020). GS is characterized by hypokalemia, hypomagnesaemia and metabolic alkalosis (Knoers and Levtchenko, 2008; Blanchard et al., 2017; Fujimura et al., 2018). DKD is associated with increased matrix expansion that manifests morphologically as a diffuse or nodular expansion of the mesangium and diffuse thickening of the glomerular and tubular basement membranes (Reidy et al., 2014; Pugliese et al., 2019).

A mutation is a change that occurs in DNA sequence either during DNA replication or as the result of exposure to environmental factors such as smoking, sunlight and radiation. A missense mutation in which a base change or substitution results in a codon that causes insertion of a different amino acid into the growing polypeptide chain, giving rise to an altered protein (Abramowics and Gos, 2018). Accumulating evidence has demonstrated that the mutations in the solute carrier family 12 member 3 (*SLC12A3*) gene, mainly including Thr60Met, Ala313Val AAsp486Asn, Gly741Arg, Arg861Cys, Leu859Pro, Cys994Tyr, Arg913Gln and Arg928Cys, cause GS (Knoers and Levtchenko, 2008; Vargas-Poussou et al., 2011; Blanchard et al., 2017; Fujimura et al., 2018; Zeng et al., 2019). Furthermore, the single nucleotide variant (SNV) Arg913Gln in the *SLC12A3* gene is found to be associated with DKD in type 2 diabetes (T2D) subjects (Tanaka et al., 2003; Nishiyama et al., 2005; Abu Seman et al., 2014; De la Cruz-Cano et al., 2019). In this review, we first summarized bioinformatics of *SLC12A3* and its genetic variation. We then described the different genetic and biological effects of the *SLC12A3* gene mutations in GS and DKD. Finally, we discuss about further genetic and biological studies of SLC12A3, and its relationship with other renal transporters in kidneys.

## Bioinformatics and Biological Function of SLC12A3

**FIGURE 1:**
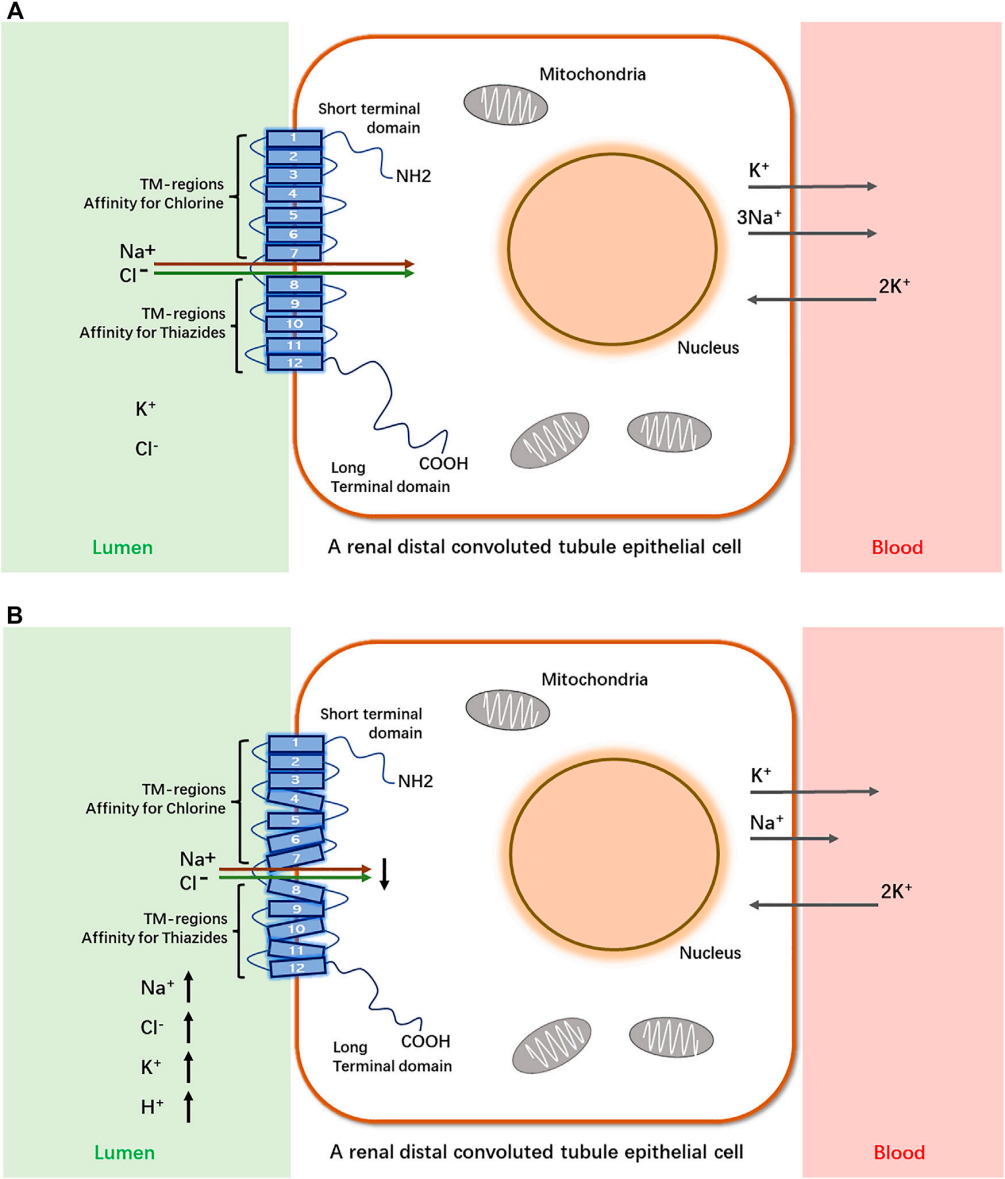
A schematic diagram of SLC12A3 as a transmembrane protein to transport Na^+^ and Cl^−^ from extra- to intra-cellular luminal sides in a renal distal convoluted tubule epithelial cell and its dysfunction in Gitelman syndrome. SLC12A3 is a protein composed of cytoplasmic topological domains and helical transmembrane regions and flanked by a short amino-terminal domain (NH2) and a long carboxyterminal domain (COOH). **(A)** In heathy subjects, most of the Na^+^ and Cl^−^ in distal convoluted tubule (DCT) of kidneys is reabsorbed by this protein. **(B)** In individuals with Gitelman syndrome, the dysfunction of SLC12A3 by inactivating mutations results in decreased reabsorption of Na^+^ and Cl^−^ in DCT. As a heterotrimer, sodium channel epithelial 1 subunits alpha, beta, and gamma (SCNN1A, B and G) in the connecting tubule (CNT) and cortical collecting duct (CCD) function with co-reabsorption of Na^+^. While SLC12A3 is dysfunctional, SCNN1 (also known as ENaC) activity is compensatory increased, resulting in a greater tubular secretion of K^+^ and other ions (Mg^2+^and Na^+^) (Colussi et al., 1997).

The kidneys are complex organs, and each human kidney has the averaged 900,000 nephrons. The nephron is basic functional and structural unit of kidneys. Structurally, the nephron consists of the glomerulus (capillaries and podocytes) located within the Bowman’s capsule and the renal tubules, including the proximal tubule, the Loop of Henle, and the distal tubule. Functionally, the nephron plays a role in the filtration and reabsorption of water and electrolytes and the secretion of wastes (Oxburgh, 2018; Kanzaki et al., 2020; Vallon and Thomson, 2020). SLC12A3 functions as a Na-Cl cotransporter and services for salt homeostasis by mediating Na-Cl transport along the renal distal convoluted tubule (DCT). Evidence from *in vivo* experimental studies has demonstrated that *slc12a3* is expressed predominantly in DCT cells in rodents (Costanzo, 1985; Ellison, 2003; Pizzonia et al., 1991; Gesek and Friedman, 1992). A schematic diagram shows that SLC12A3, as a transmembrane protein, passes through the epithelial cell membrane of renal distal convoluted tubules 12 times and physiologically functions ti transport Na and Cl ions from extra- to intra-cellular luminal sides (Figures 1A,B). In many developed countries, a high-salt diet has become an important risk factor for high blood pressure and cardiovascular diseases (CVD), and the average daily consumption has usually exceeded twice the recommended dose (5–6 g/day) (Zhao et al., 2015). SLC12A3 is sensitive to thiazide. Salt homeostasis can be affected pharmacologically by diuretic drugs (Glover et al., 2011). SLC12A3 is a good drug target, and the target mechanism is to cause salt wasting within the distal kidney nephron. By using thiazide to inhibit the *SLC12A3* gene activity, a negative sodium balance, may be induced with great health benefit in preventing hypertension and CVD. Therefore, thiazide-type diuretics are among the most widely used agents in the management of hypertension and CVD by blocking SLC12A3 (Vormfelde et al., 2003; Glover et al., 2011).

**FIGURE 2:**
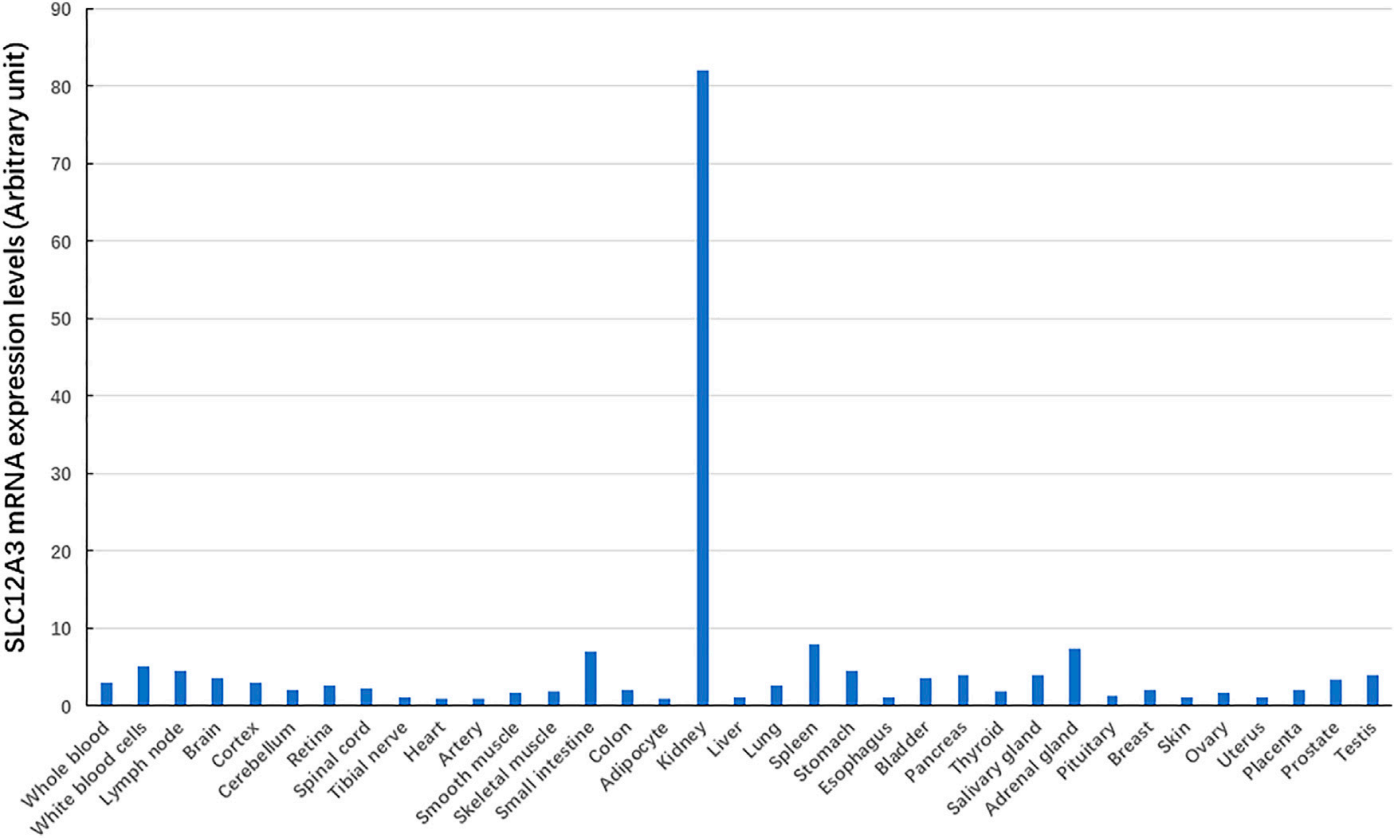
The *SLC12A3* gene expression at mRNA levels in different tissues. The *SLC12A3* gene expression at mRNA levels is predominant in kidneys, but low in adrenal gland, spleen, small intestine, and other tissues.

We have searched for bioinformatics of the *SLC12A3* gene mainly from GeneCards (https://www.genecards.org/cgi-bin/carddisp.pl?gene=SLC12A3) (Stelzer et al., 2016), and data are summarized in Table 1. Briefly, the *SLC12A3* gene is localized in human chromosome 16q13. It should be emphasized that the *SLC12A3* gene is 50,644 bp in length and consists of 26 exons. The first exon is relatively small and has a small 5′-untranslated region (UTR), while the last exon is the largest and has a large 3′-UTR. MicroRNAs (miRNAs) are endogenous RNAs and approximately 23 nt. They play important roles for gene-regulation to repress the gene activity by preferentially interacting with complementary sequence motifs in 3′-UTR of target mRNAs (Lai, 2002; Bartel, 2009). According to the information from genomic databases and literature searching, there are a total of 20 microRNAs that can regulate the *SLC12A3* gene function (Table 1). Up to date, however, there is no report concerning the regulation of these miRNAs with the *SLC12A3* gene expression in DKD except that Zhu Y et al. have reported an interaction between has-miRNA-6863 and *SLC12A3* that potentially contribute to CVD (Zhu et al., 2019). In the promoter region of the *SLC12A3* gene, there are several binding sites for transcription factors such as ATF6, E2F, GR (alpha and beta), ROR (alpha), TBP and USF1 but no CpG island exists. The *SLC12A3* gene is predominantly and highly expressed in kidneys, while mRNA expression levels of this gene in adrenal gland, spleen, small intestine, and other tissues are very low. Data are adopted from GTEx, Illumina, BioGPS, and SAGE for *SLC12A3* mRNA expression in normal human tissues (https://www.genecards.org/cgi-bin/carddisp.pl?gene=SLC12A3) and represented in Figure 2. In addition, the ortholog analysis has stated that the similarity of *SLC12A3* in mRNA sequences between human and mouse is 87.08%. The *slc12a3* mRNA expression levels are found to be over-expressed in kidneys of db/db mice from 6, 12, and 26 weeks at the age compared with the control mice at the same ages, suggesting that *SLC12A3* may play an important role not only in the kidney cloacal development but also in progress of DKD (Abu Seman et al., 2014).

**TABLE 1:** Bioinformatics of the *SLC12A3* gene.

Gene symbol	*SLC12A3*
Aliases	Solute Carrier Family 12 Member 3
	Thiazide-Sensitive Sodium-Chloride Cotransporter
	Na-Cl Cotransporter
	Na-Cl Symporter
	*NCCT*
	*NCC*
	*TSC*
ID in gene databases	HGNC: 10912
	Entrez Gene: 6559
	Ensembl: ENSG00000070915
	OMIM: 600968
	UniProtKB: P55017
Chromosomal localization	16q13
Genomic locations	chr16:56,865,207-56,915,850 (GRCh38/hg38)
	RefSeq DNA Sequence NC_000016.10
	Size: 50,644 bases; Orientation: Plus strand
Protein	Accession: P55017
	Second accessions: A8MSJ2; C9JNN9
	Size: 1021 amino acids
	Molecular mass: 113139 Da
RefSeq mRNAs	NM_000339.3; NM_001126107.2; NM_001126108.2
miRNAs	hsa-miR-136-5p; hsa-miR-335-5p; hsa-miR-515-5p; hsa-miR-519e-5p; hsa-miR-623; hsa-miR-629-3p; hsa-miR-676-3p; hsa-miR-1273e; hsa-miR-2355-3p; hsa-miR-4287; hsa-miR-4329; hsa-miR-4469; hsa-miR-4524a-3p; hsa-miR-4685-3p; hsa-miR-4780; hsa-miR-5680; hsa-miR-6867-3p; hsa-miR-6780b-3p; has-miR-6863; hsa-miR-7113-3p
Biological function	This gene encodes a renal thiazide-sensitive sodium-chloride cotransporter that is important for electrolyte homeostasis. This cotransporter mediates sodium and chloride reabsorption in the distal convoluted tubule
Diseases associated	Many mutations in this gene cause Gitelman syndrome, which is characterized by hypokalemic alkalosis combined with hypomagnesemia, low urinary calcium, and increased renin activity associated with normal blood pressure. SNV Arg913Gln in this gene is associated with diabetic kidney disease
Drug target	It is the target for thiazide diuretics that are used for treating high blood pressures

Zebrafish is a good alternative to mammalian models that can be used to apply powerful genetic experiments. In terms of kidney, zebrafish has proven itself to be the applicable and versatile experimental system, mainly due to the simplicity of its pronephros, which contains two nephrons that possess conserved structural and physiological aspects with mammalian nephrons (Morales and Wingert, 2017; Outtandy, 2019). The *slc12a3* gene in zebrafish is conserved with 62% of amino acid identity compared with the human. We have previously applied a specific morpholino oligonucleotide-mediated antisense knockdown approach in zebrafish (Li, 2017) and found that the knockdown of zebrafish *slc12a3* did not lead to global alteration of embryonic development compared to the wild-type embryos. Under the fluorescence microscopic analysis, pronephric duct epithelial structure defined by the signal (mCherry) in the cloacal portion was significantly altered at 4 dpf (Abu Seman et al., 2014). This has implicated that the *SLC12A3* gene may have important genetic effects in renal diseases.

## Genetic Effects of *SLC12A3* in Gitelman Syndrome

Gene mutation refers to random alterations in DNA that occur in somatic and reproductive cells, often during replication and division. Effects of gene mutation can range from silent expression to self-destruction. In general, point mutations are classified as missense mutations (substitutions of bases can interfere with normal protein syntheses and functioning), nonsense mutations (occur when atypical base pairings produce a stop codon that may cause improper functioning or impede functioning altogether) and frameshift mutations (occur when a nucleotide pair is added or omitted in a gene sequence that shifts how codons are read and often result in different amino acids being added to the protein being synthesized). Furthermore, recent research has underlined the abundance and importance of splicing mutations in the etiology of inherited diseases. The splicing mutation may occur in both introns and exons and disrupt existing splice sites or splicing regulatory sequences (intronic and exonic splicing silencers and enhancers), create new ones, or activate the cryptic ones (Abramowics and Gos, 2018). Up to date, application of modern techniques has allowed us to identify the genetic variants in the *SLC12A3* gene, which typically cause GS.

**FIGURE 3:**
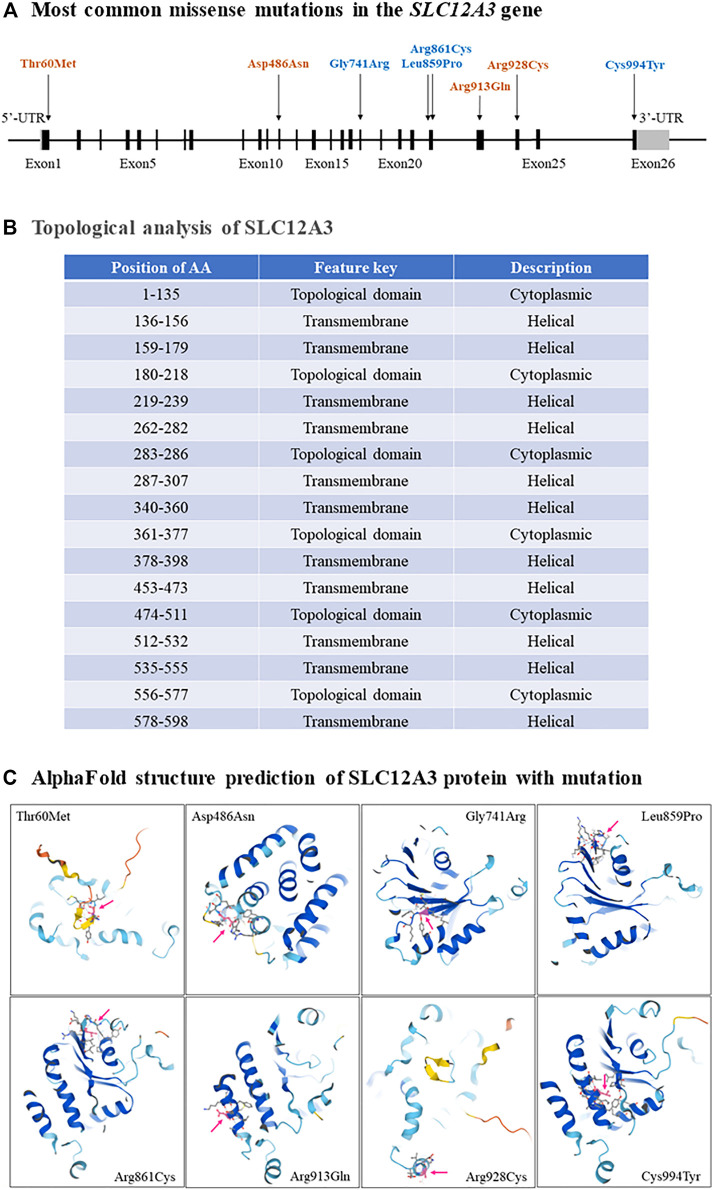
The most common missense mutations in the *SLC12A3* gene. **(A)** A schematic diagram has shown that the most common missense mutations, including Thr60Met, Asp486Asn, Gly741Arg, Leu859Pro, Arg861Cys, Arg913Gln, Arg928Cys and Cys994Tyr, are located in exons 1, 12, 18, 22, 23, 24 and 26 of the *SLC12A3* gene. **(B)** Topological analyses have indicated that all these missense mutations result in the changes of cytoplasmic topological domain but not in helical transmembrane. **(C)** The changes of SLC12A3 protein structure with each missense mutation are predicted respectively by using the AlphaFold (Jumper et al., 2021).

GS is an autosomal recessive salt-losing renal tubulopathy disorder and characterized by hypokalemia, hypomagnesemia, hypocalciuria and secondary aldosteronism (Simon et al., 1996; Lemmink et al., 1998). The major clinical characters of GS are represented in Table 2 as below. GS may be the most frequent inherited renal tubular disorders. The prevalence is estimated at ∼25 per million and accordingly, the prevalence of heterozygotes is ∼1% in Caucasian populations (Vargas-Poussou et al., 2011). The prevalence of heterozygotes in Chinese populations is ∼3%, relatively higher than what in Caucasians (Mastroianni et al., 1996; Lin et al., 2004; Hsu et al., 2009; Takeuchi et al., 2015; Zeng et al., 2019). Accumulating reports have demonstrated that many genetic variants of the *SLC12A3* gene, including missense mutations, insertion or deletion, and others such as single nucleotide variants (SNV) or repeated sequences, cause the loss of function of this gene and are responsible for the phenotypes in GS. In general speaking, point mutations in exon are categorized into missense, silent, or nonsense mutations, while missense alterations can induce the exclusion of an individual exon in various diseases. Hsu YJ et al. have analyzed DNA samples of 500 unrelated Chinese children by using PCR and restriction fragment length polymorphism approach and found that 15 mutations in the *SLC12A3* gene are associated with GS. The overall incidence of positive heterozygous mutations in the *SLC12A3* gene is 2.9%. There is no significant difference in systolic or diastolic blood pressure, biochemical profiles, or urine pH between children with heterozygous *SLC12A3* mutations and non-affected controls (Hsu et al., 2009). Takeuchi Y et al. have used a bioinformatics program to analyze 88 missense mutations in the *SLC12A3* gene and identified several mutations that may induce exon skipping in the gene (Takeuchi et al., 2015). Furthermore, Vargas-Poussou R et al. have reported that most of GS patients (70%) carry two mutations of the *SLC12A3* gene based upon sequencing analysis of genomic DNA from a large cohort of 448 unrelated patients suspected of having GS (Vargas-Poussou et al., 2011). Recently, Zeng Y et al. have reported 90 mutations in the *SLC12A3* gene based upon literature searching about Chinese patients with GS in the PubMed database and analyzing 8 GS Chinese patients (Zeng et al., 2019). Considering there is an overlap of the reported mutations in the *SLC12A3* gene among GS patients, in this review, we have summarized and represent a list of all reported *SLC12A3* genetic variants up to October 2021 in Table 3. There are a total of 150 genetic variants in the *SLC12A3* gene. Of them, 86% are missense mutations. Other variants are ins/del, SNV and CNV. In Caucasians GS patients, 5 most frequent missense mutations in the *SLC12A3* gene include Ala313Val, Gly741Arg, Arg861Cys, Leu859Pro and Cys994Tyr (blue letters in Figure 3A). Among Chinese GS patients, the most common missense mutations are Thr60Met, Asp486Asn, Arg913Gln and Arg928Cys (brown letters in Figure 3A). SLC12A3 protein (ID: P55017 in UniProtKB) is structured with six cytoplasmic topological domains and 11 helical *trans*-membranes (Figure 3B and https://alphafold.ebi.ac.uk/entry/P55017). The dysfunction of SLC12A3 in DCT, caused by SLC12A3 gene mutations, lead to GS, because the amino acid changes caused by these mutations result in the changes in the tertiary structure of the protein, and the function of the protein is reduced (Figure 3C). So far, our understanding of the changes in the functional characteristics of the most frequent *SLC12A3* mutations in GS is still limited. The main reason is that such experimental research has a certain degree of difficulty. First, different frequent mutations of different populations have indicated location and ancestral diversity of *SLC12A3* gene mutation (Maki et al., 2004; Ma et al., 2016). Second, different gene mutations can cause different changes in protein structure and function (Pandurangan and Blundell, 2020). In recent years, however, a few studies have integrated the protein configurations with the function of SLC12A3 mutations *in vitro* and *in vivo* and implicated that different mutated SLC12A3 may result in a mutation-triggered reduction in SLC12A3 protein expression, a reduction in the abundance at the plasma membrane, an impairment of protein glycosylation, and/or a disruption of phosphorylation (Yang et al., 2013; Valdez-Flores et al., 2016; Jiang et al., 2021)*.* For instance, Thr60Met is one of most frequent *SLC12A3* mutations in GS. Allele Thr60, as an important phosphorylation site, is very important for the membrane expression of SLC12A3 and phosphorylation of the adjacent Thr46 and Thr55 sites (Yang et al., 2013; Jiang et al., 2021). Furthermore, sodium channel epithelial 1 subunits alpha, beta, and gamma (SCNN1A, B and G) in the connecting tubule (CNT) and cortical collecting duct (CCD) function with co-reabsorption of Na^+^. When SLC12A3 is dysfunctional, SCNN1 activity is compensatory increased, resulting in a greater tubular secretion of K^+^ and other ions (such as Mg^2+^and Na^+^) (Hanukoglu and Hanukoglu, 2016) (Figure 1B).

**TABLE 2:** Clinical characteristics and genetic disease classification of Gitelman syndrome and diabetic kidney disease.

Disease name	Gitelman syndrome (GS)	Diabetic kidney disease (DKD)
Synonyms	Gitelman’s syndrome	Diabetic nephropathy
	Familial hypokalemia-hypomagnesemia	
Common clinical symptoms	Hypokalemic metabolic alkalosis in combination with significant hypomagnesemia and low urinary calcium excretion	Elevated blood glucose levels, increased hemoglobin A1c, and increased urinary albumin excretion
	Often muscle weakness and tetany, accompanied by abdominal pain, vomiting and fever. Paresthesias, especially in the face	Early glomerular filtration rate decline, serum uric acid; concomitant microvascular complications; and positive family history
Growth	Normal but can be delayed in the GS patients with severe hypokalemia and hypomagnesemia	Normal
Age	>6 years old and usually diagnosed during adolescence or adulthood	>40 years old and diagnosed during adulthood or old age
Blood pressures	Lower than that in the general population	Higher systolic blood pressure
Cardiology issue	Sudden cardiac arrest, occasionally	Often associated with cardiovascular diseases
Classification of genetic diseases	Autosomal recessive inherited disease	Polygenic genetic disease
Susceptibility gene	*SLC12A3*	*SLC12A3* and many others

*SLC12A3*: solute carrier family 12 (Sodium/Chloride transporter), member 3.

**TABLE 3:** Genetic variation in the *SLC12A3* gene in Gitelman syndrome.

Genetic variants		Exon
Missense mutation	Met1Leu; Thr60Met*; Asp62Asn; Glu68Lys; His69Asn; Tyr70Cys; Arg83Gln	Exon 1
	Glu131Lys	Exon 2
	Arg145His; Val153Met; Arg158Gln; Thr163Met	Exon 3
	Trp172Arg; Ser178Leu; Thr180Lys; Gly186Asp; Gly196Val	Exon 4
	Gly201Asp; Arg209Gln; Leu215Pro; Ala226Pro; Gly230Asp; Val242Ala	Exon 5
	Arg261Cys; Gly264Ala; Leu272Pro; Met279Rrg	Exon 6
	Thr304Met; Ala313Val*; Ser314Phe; Gly316Val	Exon 7
	Thr339Ile; G342A; Pro349Leu; A356V; Asn359Lys	Exon 8
	Ala370Pro; G374V; Thr382Met; Tyr386Cys	Exon 9
	Arg399Cys; Ser402Phe; Asn406His; Gly421Arg; Asn426Lys; Cys430Gly; Gly439Ser; Gly439Val; Cys421Phe; Thr428Ile; Asn442Lys	Exon 10
	Gly463Arg; Ala464Thr; Lys478Glu	Exon 11
	Asp486Asn*; Gly496Cys; Arg507Cys; Ala523Thr	Exon 12
	Asn534Lys; Phe535Leu; Leu542Pro; Phe545Leu; Ser555Leu	Exon 13
	P560H; Asn566Lys; Ala569Val; Ala569Glu; Leu571Pro; Val578Met; Aal588Val	Exon 14
	Asn611Thr; Gly613Ser; Ser615Leu; Ser615Trp; Gln617Arg; Leu623Pro; Ser628Trp; Gly630Val; His637Tyr; Asn640Ser; Arg642His; Arg642Cys	Exon 15
	Val647Met; Thr649Met; Thr649Arg; Arg655Cys; Arg655His; Arg655Leu; Val659Met; M672I; Val677Met; Val677Leu	Exon 16
	Leu700Pro; Ser710X	Exon 17
	Ala728Thr; Gly729Val; Gly731Arg; Gly741Arg*	Exon 18
	Gly800Trp; Gly800Arg	Exon 20
	Asp841Gly; Trp844X	Exon 21
	Leu849His; Leu850Pro; Arg852Cys; Arg852Ser; Leu859Pro*; Arg861Cys*; Arg861His; G867S; Arg871His	Exon 22
	Arg896Gln; Arg904Gln; Arg913Gln*	Exon 23
	Arg919Cys; Arg928Cys*	Exon 24
	Arg955Gln; Arg958Gly; Arg964Gln; S967F; Ser976Phe; Arg977X	Exon 25
	Gly980Arg; Cys985Tyr; Cys994Tyr*; Arg1009Gln; Asn1014Lys; Val1015Met; Thr1026Ile	Exon 26
Insertion/Deletion	c.234delG	Exon 1
	c.346-353delACTGATGG	Exon 2
	c.492-496delTACGGinsA	
	c.486-490delTACGGinsA	Exon 3
	c.806insTTGGCGTGGTCTCGGTCA	Exon 6
	IVS7-1G > A g.7427_7438del/insCCGAAAATTTT	Ivs7,ex8
	c.965-1 969delGCGGACinsACCGAAA	Exon 8
	c.976-977delGT	
	c.1384delG	Exon 11
	c.2454-2461delCAAGGCCC	Exon 21
	c.2850-2851delAG	Exon 24
	c.2877-2878del	
	c.2883-2884delAG	
	c.2969insGCT	Exon 26
Single nucleotide variant	c.506-1G > A	Intron 3
	c.965-2_965-1dup	Exon 7
	c.1095+4A > G	Exon 8
	c.1670-8C > T	Exon 14
	IVS16-2A > G	Ivs16,ex17
	c.2883+1G > T	Exon 24

X, stop codon; dup, duplication; * Minor allele frequency >3%.

## Genetic Effects of *SLC12A3* in Diabetic Kidney Disease

DKD has a complex etiology due to synergistic interplay among genetic, epigenetic, and environmental factors. In recent years, researchers have undertaken genetic and epigenetic studies of DKD to better understand its molecular mechanisms (Gu, 2019). Tanaka et al. have previously performed a genome-wide analysis of gene-based single nucleotide variants (SNV) in a Japanese population and reported that SNV Arg913Gln in the *SLC12A3* gene is associated with reduced risk to DKD in T2D (Tanaka et al., 2003). Another 10-years longitudinal study in the same population suggests that the 913Gln allele of this polymorphism may confer a protective effect in DKD (Nishiyama et al., 2005). After then, the association of Arg913Gln in the *SLC12A3* gene with DKD has been confirmed with a genetic study in Malaysian population (Abu Seman et al., 2014). Db/db mice are characterized by hyperglycemia, obese and urinary albumin excretion enhancement and have been widely used as a genetic rodent model for study of T2D and DKD (Sharma et al., 2003; Wang et al., 2014). Abu Seman et al. have carried out the experiments to investigate the *slc12a3* gene expression at both mRNA and protein levels in kidneys of db/db mice at the ages of 6, 12, and 26 weeks. Results demonstrate that slc12a3 expression levels at the stages of young (6 weeks old), diabetes (12 weeks old) and DKD conditions (26 weeks old) are higher than what in the control mice respectively. This implicates that *SLC12A3* is a susceptibility gene to DKD (Abu Seman et al., 2014). Furthermore, genetic studies in Japanese and Malaysian subjects with T2D-DKD demonstrate that the mutant function-loss allele 913Glu has the protective genetic effects to T2D and DKD (Tanaka et al., 2003; Nishiyama et al., 2005; Abu Seman et al., 2014). However, the association between the *SLC12A3* genetic polymorphisms and DKD is not detectable in a study with American Caucasians and possibly due to the limited statistical power in sample size (Ng et al., 2008). Furthermore, there is disagreement in the literature concerning the role of the Arg913Gln allele in diabetes and DKD, since it has been both as a predisposing and a protective allele. Two other studies in Korean and Chinese populations have considered the risk of allele 913Gln for DKD (Kim et al., 2006; Zhang et al., 2018). This disagreement is probably caused by misjudgment of the two alleles of SNV Arg913Gln in the *SLC12A3* gene but unlikely due to the population specificity.

## Summary and Perspectives

Taking together, GS is a salt-losing tubulopathy characterized by hypokalemic alkalosis with hypomagnesemia and hypocalciuria. Up to date, more than 150 mutations in the *SLC12A3* gene have been identified in GS patients. Most frequent missense mutations in the *SLC12A3* gene include Thr60Met, Ala313Val, Gly741Arg, Arg861Cys, Leu859Pro Asp486Asn, Arg913Gln, Arg928Cys and Cys994Tyr. The mutant alleles of these missense mutation are function-loss-variants. One of them, i.e. Arg913Gln in the *SLC12A3* gene is found to be associated with DKD as well. Based upon what we have described in this review, we suggest that in clinic, GS should be checked for adolescents or adults with hypokalemia, hypomagnesemia, or hypocalciuria with metabolic alkalosis, normal blood pressures. The gene detection method such as the direct sequencing of the *SLC12A3* gene and identification of the specific mutations in the gene can be performed. The next-generation sequencing technology and multiple ligation probe amplification technology have been gradually applied for diagnosis of GS (Kim et al., 2016; Ishida and Gupta, 2021).

## Abbreviation

ALPL, alkaline phosphatase, biomineralization associated; CCD, cortical collecting duct; CNT, connecting tubule; COL4A4, collagen type IV alpha 4 chain; CVD, cardiovascular diseases; DCT, distal convoluted tubule; DKD, diabetic kidney disease; GFR, glomerular filtration rate; GS, gitelman syndrome; MEFV, MEFV innate immuity regulator, pyrin; PCT, proximal convoluted tubule; S100A12, s100 calcium binding protein A12; SCNN1, sodium channel epithelial 1 subunit; SLC12A3, solute carrier family 12 member 3; SNV, single nucleotide variant; T2D, type 2 diabetes; UTR, untranslated region.
